# *Metarhizium brunneum* (Ascomycota; Hypocreales) Treatments Targeting Olive Fly in the Soil for Sustainable Crop Production

**DOI:** 10.3389/fpls.2018.00001

**Published:** 2018-01-23

**Authors:** Meelad Yousef, Carmen Alba-Ramírez, Inmaculada Garrido Jurado, Jordi Mateu, Silvia Raya Díaz, Pablo Valverde-García, Enrique Quesada-Moraga

**Affiliations:** ^1^Department of Agricultural and Forestry Sciences, ETSIAM, University of Cordoba, Cordoba, Spain; ^2^Department of Agriculture, Livestock and Fisheries, Government of Catalonia, Catalonia, Spain

**Keywords:** olive oil production, soil treatment, entomopathogenic fungi, microbial control, *Psyttalia concolor*, *Bactrocera oleae*

## Abstract

Soil treatments with *Metarhizium brunneum* EAMa 01/58-Su strain conducted in both Northern and Southern Spain reduced the olive fly (*Bactrocera oleae*) population density emerging from the soil during spring up to 70% in treated plots compared with controls. A model to determine the influence of rainfall on the conidial wash into different soil types was developed, with most of the conidia retained at the first 5 cm, regardless of soil type, with relative percentages of conidia recovered ranging between 56 and 95%. Furthermore, the possible effect of UV-B exposure time on the pathogenicity of this strain against *B. oleae* adults coming from surviving preimaginals and carrying conidia from the soil at adult emergence was also evaluated. The UV-B irradiance has no significant effect on *M. brunneum* EAMa 01/58-Su pathogenicity with *B. oleae* adult mortalities of 93, 90, 79, and 77% after 0, 2, 4, and 6 of UV-B irradiance exposure, respectively. In a next step for the use of these *M. brunneum* EAMa 01/58-Sun soil treatments within a *B. oleae* IPM strategy, its possible effect of on the *B. oleae* cosmopolitan parasitoid *Psyttalia concolor*, its compatibility with the herbicide oxyfluorfen 24% commonly used in olive orchards and the possible presence of the fungus in the olive oil resulting from olives previously placed in contact with the fungus were investigated. Only the highest conidial concentration (1 × 10^8^ conidia ml^−^) caused significant *P. concolor* adult mortality (22%) with enduing mycosis in 13% of the cadavers. There were no fungal propagules in olive oil samples resulting from olives previously contaminated by EAMa 01/58-Su conidia. Finally, the strain was demonstrated to be compatible with herbicide since the soil application of the fungus reduced the *B. oleae* population density up to 50% even when it was mixed with the herbicide in the same tank. The fungal inoculum reached basal levels 4 months after treatments (1.6 × 10^3^ conidia g soil^−1^). These results reveal both the efficacy and environmental and food safety of this *B. oleae* control method, protecting olive groves and improving olive oil quality without negative effects on the natural enemy *P. concolor*.

## Introduction

There is a need to develop effective, economically viable, and environmentally friendly methods for pest control (Nicolopoulou-Stamati et al., 2016), which has become even more critical for those insect pests that have developed insecticide-resistance such as the olive fruit fly *Bactrocera oleae* Rossi (Diptera: Tehphritidae) (Kakani et al., 2010; Hsu et al., 2015). This monophagous and multivoltine species is the most destructive to the olive crop worldwide (Daane and Johnson, 2010), not only reducing crop production, but even more important, olive oil quality (Mraicha et al., 2010; Medjkouh et al., 2016; Caleca et al., 2017). The importance of this insect pest has been aggravated by irrational repeated aerial spray applications of chemical insecticides targeting *B. oleae* adults for more than 60 years (Haniotakis, 2005). Even if most *B. oleae* control efforts have targeted the adult stage, from mid-autumn onwards, larvae of the last generation of *B. oleae* fall to the ground to pupate ~3 cm below the soil surface beneath the tree canopy, which offers a great opportunity for an effective control of *B. oleae* (Dimou et al., 2003; Ekesi et al., 2007). Faced with this scenario, microbial control of soil dwelling stages of insect pests is among the most promising alternatives to synthetic chemical pesticides (Eilenberg and Hokkanen, 2006). In particular, entomopathogenic fungi have gained importance within the entomopathogenic microorganisms mainly due to their unique contact mode of action through the cuticle (Quesada-Moraga and Santiago-Álvarez, 2008). Besides entomopathogenic fungi are naturally distributed in a wide range of habitats and the soil is considered their natural reservoir (Quesada-Moraga et al., 2007; Pell et al., 2010; Garrido-Jurado et al., 2015); therefore, soil application of entomopathogenic fungi to target soil dwelling stages of insect pests could be a powerful and sustainable pest management strategy (Rogge et al., 2017). Yousef et al. (2017) have demonstrated the efficacy of soil treatments under olive tree canopy using the *M. brunneum* EAMa 01/58-Su strain (hereafter referred to as *M. brunneum*) for *B. oleae* control, as well as the compatibility of *M. brunneum* with commercial herbicides under laboratory conditions (Yousef et al., 2015), while Garrido-Jurado et al. (2011a) have demonstrated the lack of negative direct or indirect impact of such treatments on the olive crop soil-dwelling non-target arthropod population.

The use of entomopathogenic fungi has successfully reduced by 50–70% the adult *B. oleae* spring population, showing to be an effective, economically viable, and environmentally friendly method for *B. oleae* management. However, several aspects of the method related to efficacy, food safety and environmental sustainability still need to be addressed. In this study food safety, quality, and sustainability are targeted through (1) the compatibility of *M. brunneum* strain with the herbicide Oxifourfen 24% EC that is commonly used in olive orchards in Andalusia (Spain) (2) the efficacy of this strain in different climatic conditions (North and South of Spain); (3) the effect of soil type and rainfall on the movement of conidia into the soil; (4) the effect of the exposure time to UV-B radiation on virulence of this strain against *B. oleae* adults emerging from surviving preimaginals and carrying conidia from the soil at adult emergence; (5) the effect of the fungus on the cosmopolitan parasitoid *Psyttalia concolor* (Szépligeti) (Hymenoptera: Braconidae); and (6) the presence of the fungus in the olive oil.

## Materials and methods

### Fungal strain, cultivation, and inoculum production

*Metarhizium brunneum* was obtained from the culture collection at the Agricultural and Forestry Sciences and Resources (AFSR) Department of the University of Cordoba, Spain. This strain was originally isolated from soil in a wheat plantation at Hinojosa del Duque, Cordoba, Spain. The strain was deposited in the Spanish collection of culture types (CECT) with accession number CECT 20764. The cultivation and inoculum production for the laboratory and field experiments were done as described by Yousef et al. (2017).

### Insects used in the laboratory bioassays

*Bactrocera oleae* adults used in the laboratory bioassays were obtained from naturally infested fruit collected from September to December in the Cordoba area. The infested olives were maintained as described by Yousef et al. (2013) to obtain the adults. *P. concolor* were originally obtained from a population at the Technical University of Madrid (UPM, Madrid, Spain), and then a stock colony was maintained at the Department of Agricultural and Forestry Sciences of the University of Cordoba in a rearing chamber set at 25 ± 2°C, 50–60% RH, and 16:8 h (L:D) following the standard procedure developed by Jacas and Viñuela (1994).

### Fungus-herbicide compatibility under field conditions

The field experiment was conducted in a commercial olive orchard to evaluate the compatibility of *M. brunneum* with the herbicide Oxyflourfen 24% EC. This experiment was performed in Castro del Río, Cordoba, Spain (37°41′9.5″N, 4°29′38.7″W; 227 masl). The experimental site was divided into six 1-ha square sub-fields (≈98 olive trees each; olive variety: Picual). Two of these sub-fields were the *M. brunneum*-treated plots, two were the fungi and oxyflourfen-treated plots and the others were the control plots. Soil application of the fungus was performed once in autumn (October–November) to target prepupating third-instar olive fruit fly larvae that exit from the fruits to the ground to pupate beneath the tree and spend the winter in the pupal stage (Santiago-Álvarez and Quesada-Moraga, 2007). The soil beneath each tree canopy in the olive orchards was sprayed with 1 l of *M. brunneum* suspension [which contained 1 g of conidia or 1 × 10^9^ conidia and the herbicide at recommended field concentration (2 l ha^−1^)].

To evaluate the compatibility of *M. brunneum* with the herbicide Oxyflourfen 24% EC, two types of monitoring were performed after the simultaneous treatment. The first one consisted in monitoring the fungal strain persistence in the soil from both fungus-treated plots and fungus and herbicide treated one. Before the treatment, six completely randomized soil samples were collected using a soil corer (5 cm diam) to a depth of 15 cm to determine the natural presence of indigenous entomopathogenic fungi in the soil according to Goettel and Inglis (1996). After treatment, soil samples from fungus-treated and fungus and oxyflourfen treated plots were collected monthly beneath the canopy following the same procedure as mentioned above for 6 months. To assess the conidial density in each sample, the number of colony-forming units (CFU) per gram of dry soil was determined using Sabouraud chloramphenicol agar medium in petri dishes (Goettel and Inglis, 1996). Rainfall data was obtained from the climatological stations operated by Junta de Andalucía (Red de Alerta e Información Fitosanitaria-RAIF).

In the second monitoring, the adult population dynamics was compared in both treated and control plots using a combination of pheromone yellow and McPhail traps. A total of eight traps (five yellow traps and three Mcphail traps) in each plot (treated and control) were randomly distributed and inspected weekly to count the number of *B. oleae* adults. McPhail traps were baited with diammonium phosphate 4%. To improve the count accuracy, each plot (both treated and control) was surrounded with 120 Olipe traps (one per tree) (Caballero, 2002; Altolaguirre-Obrero et al., 2003) to reduce as much as possible the entry of adults from other farms. These traps were baited with protein-based attractant, which is the most effective for the *B. oleae* (Ruiz-Torres, 2010).

### Fungal effectiveness in northern spain

The second experiment assessed the effectiveness of *M. brunneum* for *B. oleae* control under Northern Spain climatic conditions. The experiment was performed in Rodonyà located in Northern Spain (Tarragona, Spain) (41°16′38.438″N, 1°24′7.297″W; 312 masl). The experimental site was divided into four 0.5-ha square sub-fields (ca. 40 olive trees each; olive variety: Menya). Two of these sub-fields were the fungi-treated plots, and the others were the control plots. Soil application of the fungus was performed twice, once in autumn as described above and in late spring (Jun–Jul) to target the emerging adults. In both sites, the integrated pest management was applied as authorized by the Spanish Ministry of Agriculture, Food, and Environment (MAGRAMA, 2014). All treatments were performed following the same protocol described by Yousef et al. (2017).

The *B. oleae* adult monitoring described above was used to evaluate the effectiveness of the *M. brunneum* strain in soil applications in Northern Spain.

### Effect of soil type and rain volume on the movement of conidia in soil

**Figure 1:**
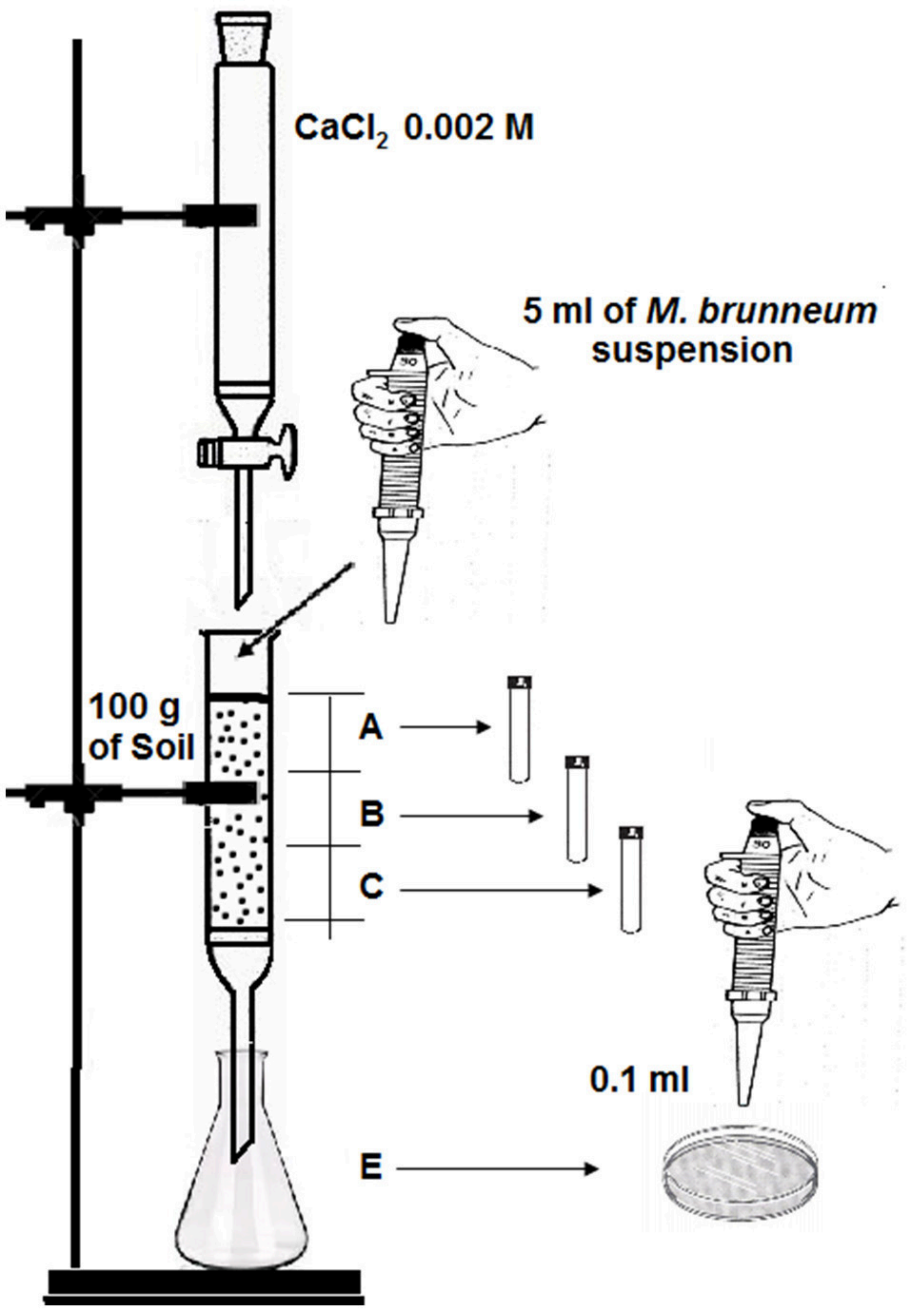
Assembly diagram to examine retention of *M. brunneum* conidia in the soil. Depth of each soil section (A, B, and C) is ca. 5 cm.

Four soils and one sandy substrate were used. The physicochemical properties of these soils are shown in Table 1. Further details of the analytical procedures used in this study have been previously described by Cañasveras et al. (2010). For the substrate preparation, quartz sand of aeolian origin was sieved to 0.2–0.5 mm, washed with a large volume of tap water enriched with Na_2_CO_3_ (pH 9.5) to disperse clay, washed with deionized water to remove salts, and finally dried in an oven at 40°C. Previously, the field capacity of each soil was estimated by saturation with water. For this purpose, five glass columns similar to a funnel (Figure 1) (140 mm height and 40 mm diameter) were packed with 100 g of soil, placing a piece of cotton in the bottom to keep the substrate in place. Deionized water was added slowly and at short intervals, ensuring that the water did not reach the lower edge of the funnel. The column was capped with Parafilm® and small holes were made with a needle. At 48 h, a portion of soil was taken from the central part of the funnel, discarding the top. In a weighing bottle of known weight (P1), the portion of soil collected was weighed (P2), placed in an oven at 105°C for 48 h, allowed to cool and reweighed (P3). Field capacity was determined using the following equation:

$$\begin{array}{l} Field\;capacity\;\left(\%\right)=\frac{P2-P3}{P3-P1}x100 \end{array}$$

To determine the influence of the volume of effluent added on the adsorption of conidia, an assembly was performed as shown in Figure 1. The five glass columns were packed with 100 g of sterile soil (autoclaved for 60 min twice at an interval of 24 h) representing model combinations of texture (sandy or clay) and pH (acid or alkaline), a piece of cotton was placed in the bottom to keep the substrate in place. Then, 5 ml of a 10^8^ conidia/ml suspension was added to the soil surface. The columns were then run with the test volume (i.e., 60, 85, 100, 140, 250, 300, or 400 ml) of 0.002 M CaCl_2_, added dropwise with a crystal burette. At the bottom, an Erlenmeyer flask was placed to collect percolated effluent and the collected volume was recorded.

**Table 1:** Geographical location and physicochemical properties of the soil samples used in this work.

**Soil name**	**Geographical location**		**Soil order**	**Soil factors**										
	**Locality**	**Province**		**Sand**	**Silt**	**Clay**	**Textural class**	**OM**	**CaCO_3_**	**pH**	**EC 1:5**	**CEC**	**Fe_d_**	**DC**
				**(g/kg)**	**(g/kg)**	**(g/kg)**		**(g/kg)**	**(g/kg)**		**(μS/cm)**	**(Cmol_c_/kg)**	**(g/kg)**	**(g/kg)**
AG35	Pozoblanco	Córdoba	Alfisol	860	90	50	Sandy	14	2	6.0	17	7	3	23
AG51	La Luisiana	Sevilla	Alfisol	400	120	480	Clay	6	0	6.3	402	8	25	153
AG53	SantaCruz	Córdoba	Vertisol	159	250	592	Clay	5	215	8.5	105	13	5	187
INM9	Fuente de Piedra	Málaga	Alfisol	545	255	200	Sandy clay loam	24	674	8.4	330	9	4	124

*OM, organic matter; EC, electrical conductivity; CEC, cation exchange capacity; Fe_d_, dithionite extractable iron (Mehra and Jackson, 1958); DC, dispersable clay*.

To determine the number of CFU, each column was divided into three equal sections or depths (A, B, and C; ca. 5 cm each one). 1 g of soil from each section was dissolved in 10 ml of deionized water with Tween 80 (0.1% v/v) and shaken at 12 rpm in a Model 3000445 Orbit rotary stirrer (J.P. Selecta, Barcelona, Spain) for 90 min. Aliquots of 0.1 ml were then spread with a Drigalsky loop on Saboraud dextrose agar supplemented with 0.5 g l^−1^ of chloramphenicol (SDAC, Biolife, Italy). Aliquots of 0.1 ml of the collected effluent (E) were also spread on the SDAC medium. In some cases, dilutions were necessary before spreading. The plates were cultured at 25°C for 3–4 days and CFU were counted. Fungal growth was visually identified: *M. brunneum* colonies exhibited circular growth, were largely white and contained varying shades of green in the central mycelia (Humber, 1997).

### Effect UV-B radiation exposure time on the pathogenicity of *M. brunneum* against *B. oleae* adults

The irradiation experiment was conducted in a temperature-controlled chamber (Fitoclima S600PL, ARALAB, Portugal) at a constant temperature of 25 ± 1°C. To filter out the radiation below 290 nm, the irradiated samples (UV-B treatments) were protected with a 0.13 mm-thick cellulose-diacetate film (Clarifoil, Texas, USA), which allowed passage of most of the UV-B and UV-A radiation but prevented the UV-C radiation (<280 nm). In parallel a “no UV-B treatment” was performed, where cages were wrapped in aluminum foil to prevent UV-B exposure during the irradiation.

A *M. brunneum* conidial suspension (10^9^ conidia ml^−1^) was prepared. Then, newly emerged (24 h) *B. oleae* adults were cold anesthetized and treated with 1 ml of conidial suspension by using a Potter Spray Tower (Burkard Rickmansworth Co, Rickmansworth, UK). There were two controls, the first one was treated with the same volume of a sterile 0.1% Tween 80 aqueous solution and then irradiated, and the other was treated with the fungus and not irradiated. Following treatment, the insects were placed in the above-mentioned methacrylate cages. Then, the UV-B treatment (inoculated with the fungi and covered with cellulose-diacetate film) was irradiated at 1200 mW m^−2^ for 2, 4, or 6 h. The no UV-B treatment (inoculated with the fungi and covered with aluminum foil) was irradiated for 6 h. Adult diet and water were provided *ad libitum*. Three replicates of 10 insects each were used for the UV-B and no UV-B treatments. Mortality was monitored for 10 days. Dead flies were removed daily and processed to assess mycosis.

### Virulence assay of *M. brunneum* against *P. concolor* adults

Virulence of *M. brunneum* against *P. concolor* adults was evaluated under laboratory conditions. Four *M. brunneum* concentrations were prepared in a sterile aqueous solution that contained 0.1% Tween 80 [10^5^, 10^6^, 10^7^, and 10^8^ conidia ml^−1^], with the control consisting of no conidia. Newly emerged *P. concolor* adults that had previously been cold-anesthetized were treated with a Potter Spray Tower. Half a milliliter of conidial suspension was used for each replicate, with three replicates per treatment (10 adults each). After the treatment, adults were placed in methacrylate cages (80 × 80 × 60 mm, Resopal, Madrid, SP) with lids containing a 20 mm diam circular hole covered with a net cloth. Adult (treated and control) parasitoids were fed on honey. Mortality data were registered for 10 days. Dead flies were removed daily and the development of mycosis on the cadavers was determined.

### Evaluation of the presence of fungal structures in olive oil

To assess the possible presence of *M. brunneum* conidia in olive oil after the treatment, two laboratory experiments were performed In the first one, the olives were directly exposed to the fungus, creating a “worst case” scenario. For that, three olive samples (1 kg each) were placed on trays (690 cm^2^) and sprayed with 5.75 ml of 10^9^ conidia ml^−1^ with an Aerograph 27085 (piston compressor of 23 l min^−1^, 15–50 PSI, nozzle diameter of 0.3 mm, China). After treatment, olives remained in the trays for 48 h, and then olives from different trays were mixed and processed in three different groups before milling: (1) olives from the first group were rinsed in water for 1 min; (2) olives from the second group were surface-sterilized with 1% sodium hypochlorite; and (3) olives from the third group remained unwashed. Finally, olives from each group were processed into oil following the extraction process described by Vossen (2007) with some modifications. Olives were crushed for 2 min at 37°C using a Thermomix® TM31 (Vorwerk, Wuppertal, D). After grinding, the paste was warmed in the same Thermomix for 30 min at 27.5°C. Then, the oil was extracted through combination of pressing and centrifuge rotating at ~10000 rpm.

In the second experiment, the olives were indirectly exposed to the fungus, creating a “real case” scenario for *M. brunneum* soil application. The above-mentioned trays were prepared with 500 g of soil that covered the entire base. The soil used in this study was collected from a farm in Córdoba and was characterized as sandy loam (78.0% sand, 17.0% silt, 5.0% clay, and 0.2% organic matter) with a pH of 8.3. The soil was sieved (2-mm mesh) and stored in a dry place at ~25°C. The soil was then sterilized at 121°C for 20 min and dried in an oven at 105°C for 24 h. Then, soils were sprayed with 5.75 ml of the fungal suspension (10^9^ conidia ml^−1^) using the above-described aerograph. After treatment, soil samples were collected from the bioassay trays. The conidial density after treatment was assessed using the CFU per gram of dry soil. Then, olives were dropped onto soil (treated and control). Olive samples were taken from the trays every 4 days for 16 days. Three replicates (trays) per sampling date were used for treatment and control. Finally, olive oil of each sampling date was extracted following the above-described protocol.

For both experiments, the possible presence of the fungus was evaluated according to the CFU method: 100 μl of each oil sample was spread onto medium and incubated at 25°C for 15 days.

### Statistical analysis

The area under the *B. oleae* flight curves (AUBFC) in treated and control plots was calculated by trapezoidal integration method of SAS (Campbell and Madden, 1990). Then, the values of AUBFC were log_10_ transformed and subjected to factorial analysis of variance using Statistix 9.0 (Analytical Software 2008). The same program was used to analyze mortality data. The values of average survival times obtained by the Kaplan-Meier method and compared using the log-rank test were calculated with SPSS 15.0 Software for Windows. Replicates in time, for all experiments, were analyzed as series of experiments with the model, *y* = *treatment* + *experiment* + *treatment* × *experiment* (Littell et al., 2006). Since the effect of *experiment* and interaction *treatment* × *experiment* was not significant, replicates from both experiments were combined in a model with only *treatment* as a factor (one-way ANOVA). Mortality data was transformed, $y=\arcsin\left(\sqrt{\frac{\%\;Mortality}{100}}\right)$, to improve normality and homogeneity of variance, both requirements for linear model analysis. Means from different treatments were compared using Tukey's test (*P* < 0.05). The effect of soil type and rain volume on relative percentage of *M. brunneum* recovered was evaluated with a generalized linear model for ordinal data (proportional odds model). This proportional odds model is the standard generalized linear model for ordinal regression (Stroup, 2012) and is appropriate for this experiment since we are measuring the response as ordinal data type, expressed as relative or cumulative percentage of conidia in each section of the soil column. The dependent variable or response is the four possible classes or soil sections (A, B, C, and E). This model calculates the cumulative probability or proportion of conidia at each soil section or in the sections above. i.e., *P* (*conidia* ≤ *A*) is the probability or proportion of conidia in section A; *P* (*conidia* ≤ *B*) is the probability or proportion of conidia in sections A and B; *P* (*conidia* ≤ *C*) is the probability or proportion of conidia in sections A, B, and C; and *P* (*conidia* ≤ *E*) is the probability or proportion of conidia in the effluent (E) or in any of the sections = 100%. The proportion of conidia in one specific section can be calculated then as the difference between contiguous cumulative probabilities e.g., probability or proportion of conidia in section B, *P* (*conidia* = *B*) = *P* (*conidia* ≤ *B*) − *P* (*conidia* ≤ *A*). The equation of the model is:

$$\begin{array}{l} \eta_{cijk}=\eta_{c}+{Soil\;type}_{i}+{Rain\;volume}_{j}+Soil\;type\\ \;{\times Rain\;volume}_{ij},\;c\;\left(class\right)=A,B,C,E \end{array}$$

Where: Rain volume is modeled as continuous factor or covariate; k sub index refers to replicate; observations follow a multinomial distribution; and cumulative logit link function.

For the generalized linear model the estimation method was maximum likelihood with Laplace approximation. Model significance was evaluated with χ^2^ test and the significance of the fixed effects was evaluated with F-approximate test (α = 0.05). Estimated cumulative probabilities for soil types were compared with odds ratio test. If the confidence interval for the ratio includes 1, the two soils are not significantly different (Stroup, 2012).

## Results

### Fungus-herbicide compatibility and fungal effectiveness in northern spain

**Figure 2:**
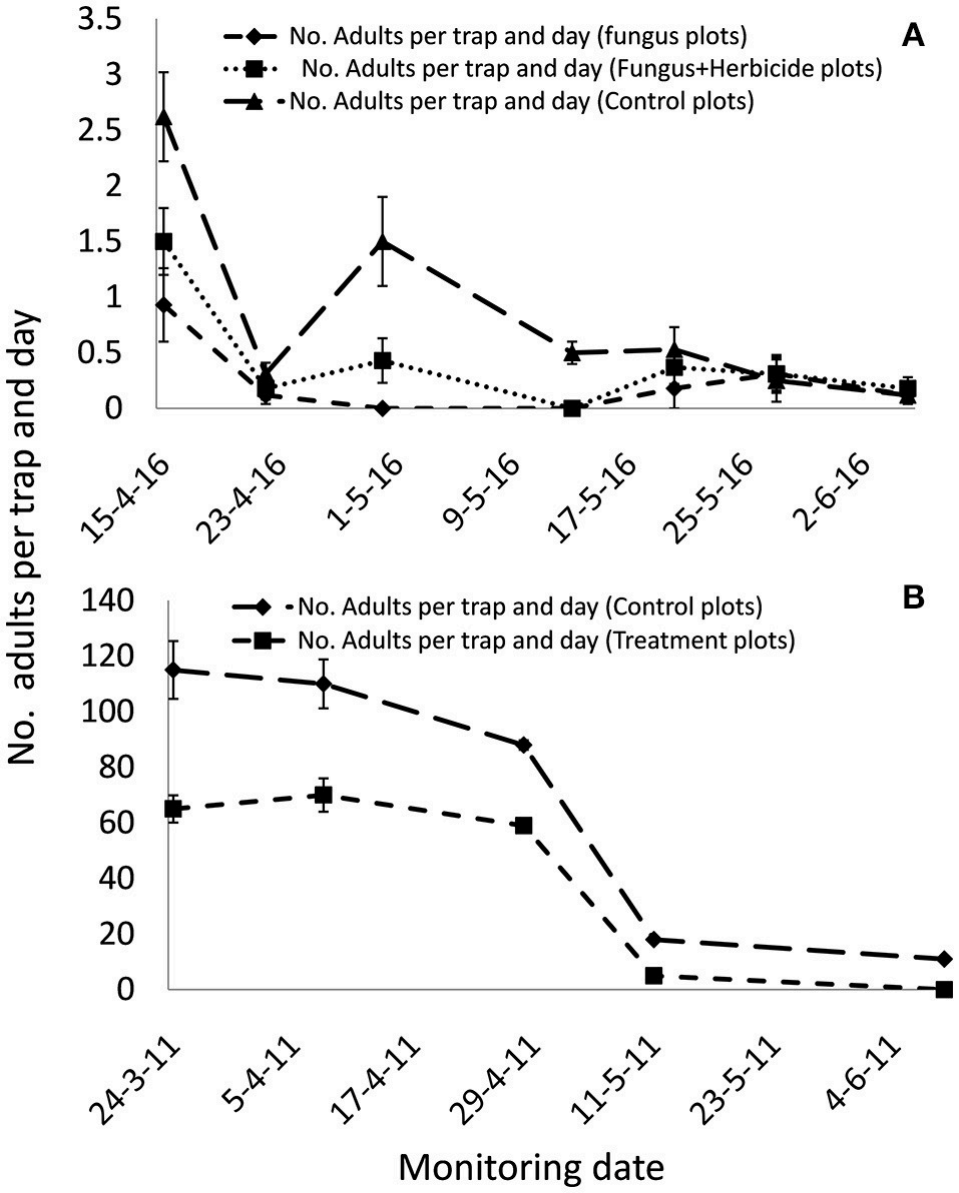
*Bactrocera oleae* population densities emerged from soil in treated and control plots after *M. brunneum* soil application underneath the tree canopy. **(A)** Fungus-herbicide compatibility bioassay performed in Southern Spain with simultaneous application of the fungus with the herbicide Oxyflourfen 24%EC. **(B)** Fungus efficacy under Northern Spain climatic conditions.

**Figure 3:**
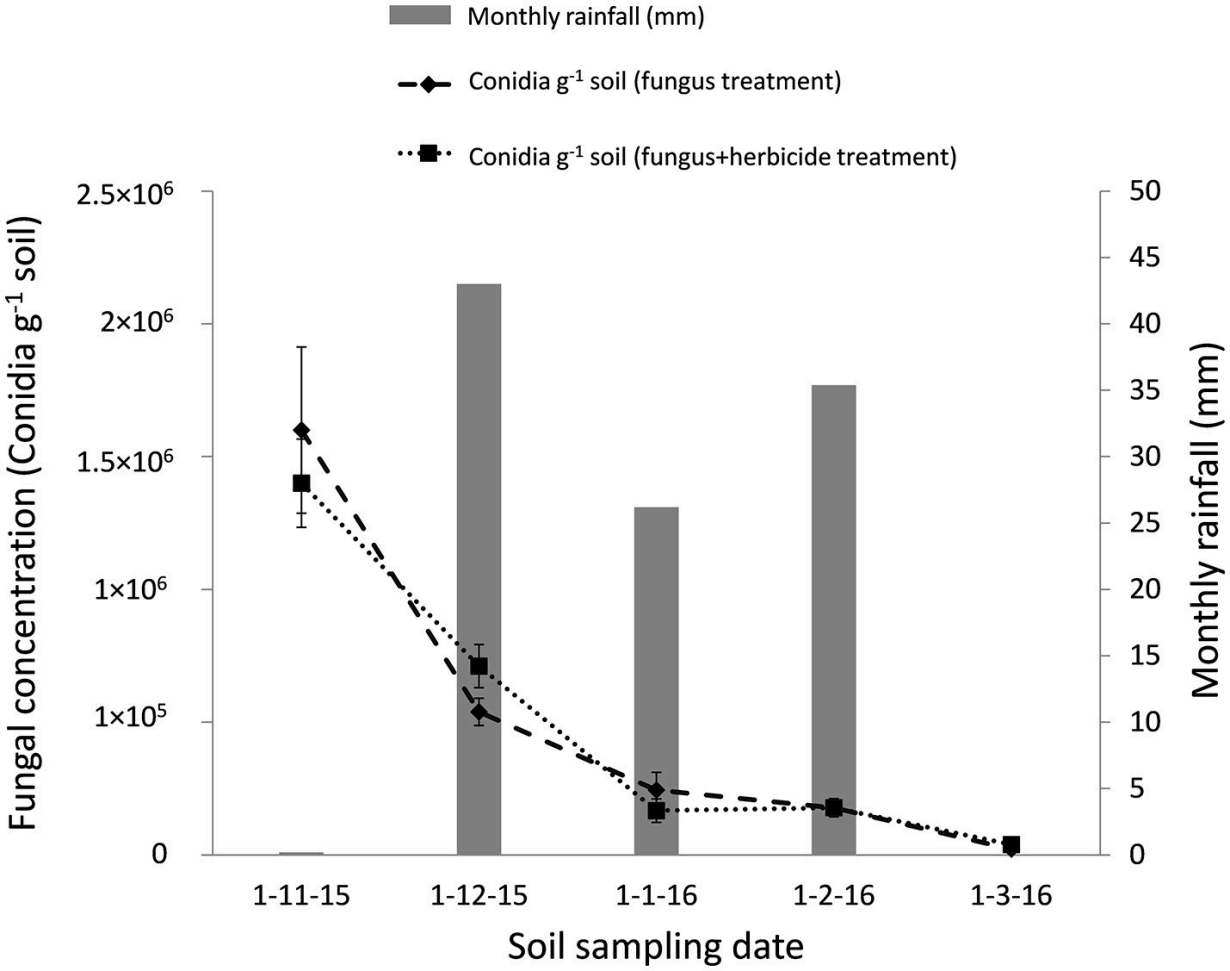
Time course of *M. brunneum* concentration in the soil (Southern Spain) after both fungus treatment and fungus-herbicide simultaneous treatment and monthly rainfall during the period.

The soil applications of *M. brunneum* in both Northern and Southern Spain reduced the *B. oleae* population in treated plots compared with controls (Figure 2). The reduction in the *B. oleae* adult population emerging from the treated plots compared to the control plots was significant even if the fungus mixed with the herbicide (*P* < 0.5). In the Southern Spain experiment, the maximum captures of adult flies was 0.9 and 1.5 flies per trap/day in the fungus and fungus and herbicide treated plots, respectively, compared with 2.6 flies per trap/day in the control plots. In Northern Spain, the maximum captures of adult flies were 65 flies per trap/day in the fungus treated plots compared with 115 flies per trap/day in the control plots. The *M. brunneum* soil density decreased after autumn treatments in relation with the monthly rainfall and date of fungus application (Figure 3). However, the fungus persisted in the soil in both fungus and fungus and herbicide treated plots. The fungal concentrations in the soil 4 months after treatment in both fungus and fungus and herbicide treated plots were 1.2 × 10^3^ and 2.3 × 10^3^ conidia per gram of soil, respectively (Figure 3).

### Effect of soil type and rain volume on the movement of conidia in soil

The analysis of the effect of soil type and rain volume on relative percentage of *M. brunneum* recovered showed a significant effect of soil type [*F*_(4, 130)_ = 33.64, *P* < 0.0001], rain volume [*F*_(1, 130)_ = 390.80, *P* < 0.0001], and interaction soil type x rain volume [*F*_(4, 130)_ = 8.42, *P* < 0.0001]. The whole model was significant $\chi_{9df}^{2}=$ 2347.08, *P* < 0.0001.

The effect of rain volume on the relative percentage of conidia recovered for sections from A to E was inversely proportional for the series AG51, AG53, AG35, INM9, and FOCS.

**Figure 4:**
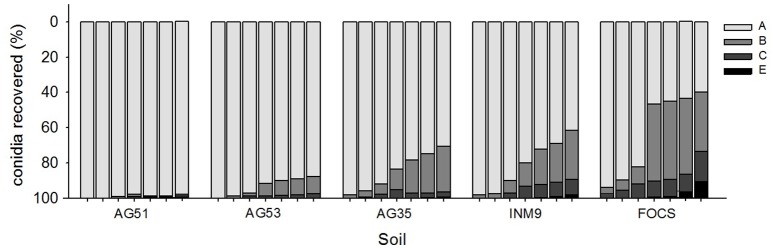
Effect of rain volume on the relative percentage of conidia recovered for soil sections from A to E in each substrate (Figure 1). Columns within each soil class represent the different amounts of rain evaluated in this experiment (60, 85, 100, 140, 250, 300, and 400 ml).

The soils with more retention in the first section (A) were the clayey soils AG51 and AG53, with relative percentages of conidia recovered ranging between 83 and 97% of conidia, for the range of water volume evaluated. There were no significant differences between the profiles of retention for these two soil types (odds ratio test, 95% CI = 0.96–2.77) (Table 2 and Figure 4). The retention in the first section significantly decreased in the sandy soils AG35 and INM9 in comparison with the clayey soils AG51 and AG53. The relative percentage of conidia in section A ranged between 55 and 91%, with cumulative values for sections A and B ranging between 85 and 98%. There were no significant differences between the profiles of AG35 and INM9 (odds ratio test, 95% CI = 0.77–1.44). The soil type with significant lower retention in the first sections was FOCS, with 23.4% of conidia in section C and effluent of 9.1% at the maximum rain volume of 400 ml.

**Table 2:** Relative percentages (cumulative values) of conidia at different soil sections and soil types.

**Soil type**	**Rain volume (ml)**	**Soil section A relative % (cumulative)**			**Soil section A** + **B relative % (cumulative)**			**Soil section A + B + C relative % (cumulative)**			**Comparison of soil types odds ratio test**
		**Estimate**	**Low 95%**	**Upp 95%**	**Estimate**	**Low 95%**	**Upp 95%**	**Estimate**	**Low 95%**	**Upp 95%**	
AG51	140	97.2	96.4	97.8	99.3	99.2	99.5	99.8	99.8	99.8	a
AG51	400	95.3	93.4	96.7	98.9	98.4	99.2	99.7	99.6	99.8	
AG53	140	94.0	92.9	94.9	98.6	98.3	98.8	99.6	99.6	99.7	a
AG53	400	83.5	80.3	86.3	95.8	94.8	96.6	99.0	98.7	99.2	
AG35	140	88.2	86.8	89.5	97.1	96.7	97.5	99.3	99.2	99.4	b
AG35	400	66.1	62.4	69.7	89.8	88.1	91.3	97.5	96.9	97.9	
INM9	140	86.2	84.6	87.6	96.6	96.1	97.0	99.2	99.0	99.3	b
INM9	400	55.9	52.0	59.7	85.2	83.0	87.1	96.2	95.3	96.8	
FOCS	140	71.9	70.0	73.8	92.1	91.2	92.8	98.0	97.7	98.3	c
FOCS	400	31.3	28.3	34.5	67.4	64.2	70.5	90.1	88.3	91.6	

*Values estimated with the generalized linear model for ordinal data for the exemplary cases of rain volume = 140 and 400 ml. Depth of each soil section from A to C is ca. 5 cm*.

### Effect UV-B radiation exposure time on the virulence of *M. brunneum* EAMa 01/58-Su strain against *B. oleae* adults

The virulence of *M. brunneum* was slightly higher on non-irradiated insects than UV-B treatment, with mortality ranging from 16.6 (non-treated and non-irradiated adults) to 92.9% (treated and non-irradiated adults), and fungal outgrowth from cadavers reaching 73.3% (Table 3). However, the average survival time (AST) of fungus-treated and irradiated adults were statistically equal to the AST of fungus-treated and non-irradiated adults, both, statically lower of AST of non-treated and irradiated adults or non-treated and non-irradiated adults (Table 3).

**Table 3:** Effect of the exposure time to UV-B (1200 mWm^−2^) on the virulence of *M. brunneum* strain against adult *B. oleae*.

**Treatment^a^**	**Kaplan-Meier survival analysis**		**Mortality % (mean ± SE)**	
	**AST (days)^b^**	**95% FL**	**Total**	**With fungal outgrowth**
2 h	9.0 ± 0.2a	8.5–9.5	90.0 ± 5.7a	73.3 ± 12.0a
4 h	9.0 ± 0.2a	8.5–9.6	79.2 ± 5.8a	60.0 ± 10.0a
6 h	9.0 ± 0.3a	8.4–9.6	76.7 ± 7.3a	56.6 ± 12.0a
Control 1	10.6 ± 0.4b	9.8–11.4	20.0 ± 15.2b	0b
Control 2	8.7 ± 0.2a	8.1–9.2	92.9 ± 3.5a	63.3 ± 12.0a
Control 3	10.3 ± 0.3b	9.5–1.0	16.6 ± 6.6b	0b

a *Control 1, Adult B. oleae were sprayed with 1 × 10^9^ conidia ml^−1^ and placed in methacrylate boxes, which were covered with cellulose-diacetate film (UV-B treatment) and aluminum foil for 6 h; Control 2, Treated and non-irradiated insects placed inside the chamber for 6 h; Control 3, non-treated and non-irradiated insects placed inside the chamber for 6 h*.

b *AST (mean ± SE) limited to 10 days. Data in the same column followed by the same letter are not significantly different (α = 0.05) according to the log-rank test*.

### Virulence assay for *M. brunneum* against *Psyttalia concolor* adults

**Figure 5:**
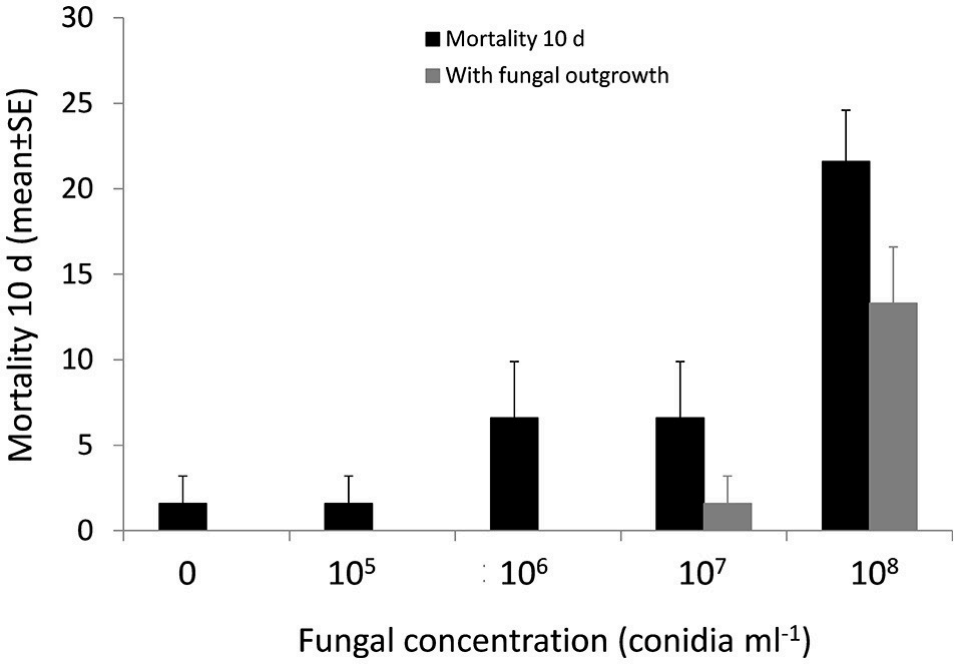
Percentage mortality and fungal outgrowth (mean ± SE) of newly emerged *P. conocolor* adults after 10 days of exposure to suspensions of conidia at different concentrations.

*P. concolor* adult mortality due to *M. brunneum* differed significantly at different conidial concentrations (*P* < 0.001). However, only the highest concentration (10^8^ conidia ml^−1^) caused significant *P. concolor* adults mortality (21.6%) (Figure 5). *P. concolor* adult mortality caused by *M. brunneum* ranged between 1.6 and 6.6% at 10^5^ to 10^7^ conidia ml^−1^ compared with control mortality of 1.6%. Furthermore, the fungal treatment significantly influenced mycosis on the cadavers (*P* < 0.001), with mycosis on 13.3, 1.6, 0, and 0% of the cadavers treated with 10^8^, 10^7^, 10^6^, and 10^5^ conidia ml^−1^, respectively. Only the highest concentration (10^8^ conidia ml^−1^) was statically different from the control (Figure 5).

### Evaluation of the presence of fungal structures in the olive oil

No fungal propagules were detected in any of the olive oil samples obtained from both olive experiments, in which olives were directly or indirectly exposed to the fungus. There were no CFU in any of the Petri plates inoculated with the different olive oil samples.

## Discussion

Throughout the last decades the development of biopesticides based on entomopathogenic fungi has become a very active line of work, since these products constitute an environmentally friendly alternative to synthetic/chemical pesticides (Sinha et al., 2016). Entomopathogenic fungi have been mainly investigated for aboveground pest control, whereas their potential for the control of soil-dwelling pests has been mostly ignored (Jackson et al., 2000). We had already developed a pioneer method based on soil application of *M. brunneum* for olive fruit fly control by targeting third-instar larvae in the soil during autumn and emerging adults from the soil during spring (Yousef et al., 2017). However, the results of the present work present additional evidence of the potential of this strategy to develop a safe and sustainable olive pest IPM.

*Metarhizium brunneum* mixed with the herbicide Oxyflourfen 24% EC in the atomizer tank and then applied beneath the olive trees canopy, reduced the *B. oleae* population that emerged during spring from the soil of treated plots compared to controls plots in the range of 43% (fungus+herbicide treatment) and 65% (fungus alone treatment). This *in vivo* compatibility of *M. brunneum* with the herbicide confirms our previous laboratory results (Yousef et al., 2015), and allows a fungus-herbicide simultaneous application reducing the application costs of both the fungus and herbicide. In addition, it is noteworthy that this reduction in *B. oleae* population density was a result of a single *M. brunneum* soil application in autumn. However, in our previous study, the soil application was performed twice, targeting preimaginals in the soil in autumn and targeting adults at emergence from the soil in spring, providing between 50 and 70% of reduction in *B. oleae* population density (Yousef et al., 2017). The persistence of the fungus in the soil after treatment was not affected by the herbicide mixed with the fungus, with *M. brunneum* persisting in the soil, after both fungus treatment and fungus-herbicide simultaneous treatment during 4 months at the onset of the natural background concentration registered in bulk soil (Bruck, 2010; Scheepmaker and Butt, 2010). This is noteworthy because the decline of entomopathogenic fungi densities over time to acceptable background level is requested by the EU regulations for registration purpose (Scheepmaker and Butt, 2010). This gradually decrease in time of the entomopathogenic fungi density in the soil may be influenced by edaphic, biotic, climatic, and cultural factors (Quesada-Moraga et al., 2007; Scheepmaker and Butt, 2010; Garrido-Jurado et al., 2011b). The effect of rainfall in the fate of fungal propagules in soil is the least understood, and has been shown to affect the foliar use of entomopathogenic fungi or the dispersion in soil, but in relation to crop residue (Bruck and Lewis, 2002; Jaronski, 2010). Many studies have suggested that rainfall is an important abiotic factor affecting conidia vertical mobility in the soil (Inglis et al., 2001; Garrido-Jurado et al., 2011b). However, this is first time that the fate of the fungal inoculum along the soil profile has been examined after the soil has been treated with entomopathogenic fungi. The results of the present work may serve as a model useful in estimating the fungal inoculum contained in each soil section according to soil type and amount of rain. More than 50% of the conidia was retained in the first 5 cm of the soil regardless of soil type and rain amount, which would guarantee the contact of the fungal propagules with *B. oleae* preimaginals in the soil since around 80% of the third instar prepupating olive fruit fly larvae pupate ~3 cm below the soil surface (Dimou et al., 2003).

These results reveal both the high potential of *M. brunneum* and the key impact of the autumn soil application for the control of *B. oleae*. Our study validates the effectiveness use of *M. brunneum*-soil applications as a pest management method in Northern Spain since both the climatic conditions and biological activity of *B. oleae* (up to 5 generations per year) are different to those in Southern Spain (Santiago-Álvarez and Quesada-Moraga, 2007). Surprisingly, similar results were obtained with a reduction percentage of almost 50% in the *B. oleae* population emerged from the soil of treated plots during spring compared with controls.

Our results also reveal that UV-B did not reduce the virulence of *M. brunneum* against *B. oleae* when adults were irradiated at 1200 mW m^−2^ for 2, 4, and 6 h after being treated with the fungus. Even if the *B. oleae* adult mortality has been slightly reduced after 6 h exposure to UV-B, the AST of fungus-treated and irradiated adults were statically equal to the AST of fungus-treated and non-irradiated adults. However, the fungus applied to the soil during autumn targeting *B. oleae* larvae will be protected from environmental conditions, since the soil is the main natural reservoir for native entomopathogenic fungi and provides protection from extreme conditions (Ekesi et al., 2007; Zimmermann, 2007a,b; Garrido-Jurado et al., 2015). Nevertheless, it is interesting to know how UV-B may affect fungal virulence in *B. oleae* adults that emerge from the soil during spring from surviving preimaginals. Generally, UV-B, can be particularly deleterious for entomopathogenic fungi due to its detrimental effects on various fungal biological processes (Inglis et al., 2001; Jaronski, 2010). Fernández-Bravo et al. (2017) have shown that UV-B radiation may potentially affect the virulence of *M. brunneum* against *Ceratitis capitata* adults, but the decrease of the conidial density due to UV-B exposure was not enough to avoid a viable number of conidia remaining that exceeded the threshold to cause disease.

Once again, *M. brunneum* was shown to be safe for *P. concolor* in olive orchards. The highest *P. concolor* adult mortality caused by this strain was 21.6% at a concentration of 10^8^ conidia ml^−1^. However, the same concentration (10^8^ conidia ml^−1^) has caused more than 95% of *B. oleae* adult's mortality (Yousef et al., 2017). Nevertheless, at field conditions, the direct contact between *M. brunneum* applied to the soil and the parasitoid is not possible. Furthermore, studies of Ekesi et al. (2005) and Daane et al. (2015) have demonstrated that fungal application targeting tephritid fruit fly preimaginals in the soil can be compatible with the classic biological control in which *Psyttalia* spp. larval parasitoids are field released. Our previous studies have shown that soil applications of *M. brunneum* are safe to soil dwelling non-target arthropod communities, specially the formicid *Tapinoma nigerrimum* (Hymenoptera: Formicidae) used as a bioindicator in olive groves (Garrido-Jurado et al., 2011a).

All olive oils obtained from olives previously exposed to *M. brunneum* were free of fungal propagules. The possibility of direct contact between the fungus and the olives is minimum, in spite of the direct contact experiment as a “worst case” scenario or the real conditions (soil application of the fungus). To date, this is the first study that addresses such possible presence of fungal inoculum in oils obtained from olives exposed to entomopathogenic fungi. These results demonstrate the food safety of this control method compared to the chemical insecticides used for more than 60 years for *B. oleae* control (Haniotakis, 2005), since pesticide residues have been detected in oils obtained from treated olives (Lentza-Rizos and Avramides, 1995). In addition, olive oil extraction at different exposure time to *M. brunneum* shows that there is no minimum waiting period between the application of the fungus and the harvest of the fruit.

This work highlights the adaptation of the olive fruit fly control method based on soil application of *M. brunneum* to the different climatic conditions and the possibility of a simultaneous soil application of the fungus with the herbicide beneath the tree canopy, which reduces application costs. This, together with the absence of negative effects on *P. concolor*, a *B. oleae* cosmopolitan parasitoid, demonstrates the environmental sustainability of this innovative method of control. In addition the use of entomopathogenic fungi leaves no residues in olive oil, in contrast to the use of chemical insecticides.

## Author contributions

MY, IG, and EQ-M designed the experiments. MY, CA-R, SR, and JM performed the experiments. MY, and PV-G analyzed the data. MY, IG, and EQ-M wrote the manuscript.

### Conflict of interest statement

The authors declare that the research was conducted in the absence of any commercial or financial relationships that could be construed as a potential conflict of interest.
